# First description of a cefixime- and ciprofloxacin-resistant *Neisseria gonorrhoeae* isolate with mutations in key antimicrobial susceptibility–determining genes from the country of Georgia

**DOI:** 10.1016/j.nmni.2018.04.004

**Published:** 2018-04-25

**Authors:** M.A. Washington, A.E. Jerse, N. Rahman, M. Pilligua-Lucas, E.C. Garges, N.H. Latif, T. Akhvlediani

**Affiliations:** 1) US Army Medical Research Directorate–Georgia (USAMRD-G), Tbilisi, Georgia; 2) Uniformed Services University of Health Sciences (USUHS), Bethesda, MD, USA

**Keywords:** Antimicrobial, Georgia, gonorrhoea, mutation, *Neisseria*, resistance

## Abstract

Antimicrobial resistance in *Neisseria gonorrhoeae* is a global health problem. Enhanced international collaborative surveillance and disease control are needed to reduce the global burden of this important pathogen. Currently the antimicrobial resistance properties and molecular mechanisms of multidrug-resistant *N. gonorrhoeae* in the Republic of Georgia represent a significant knowledge gap. Here we report the isolation of a strain of *N. gonorrhoeae* exhibiting resistance to cefixime and ciprofloxacin with reduced susceptibility to penicillin and tetracycline from a patient being treated at a Georgian medical centre. Notably, this isolate was found to contain a mosaic *penA* allele and to harbour mutations in genes conferring susceptibility to the β-lactam, cephalosporin, fluoroquinolone, macrolide and penicillin classes of antibiotic. To our knowledge, this is the first report to describe the key mutations conferring the antimicrobial resistance properties of an isolate of *N. gonorrhoeae* from Georgia.

## Introduction

Historically, *Neisseria gonorrhoeae* has been associated with a high rate of morbidity in military populations [1], [2]. Soldiers with urogenital infection can enter into a state of debilitation preventing them from performing their duties [3]. Importantly, untreated or incompletely treated *N. gonorrhoeae* infection can lead to significant and potentially life-threatening sequelae including pelvic inflammatory disease, epididymitis and infertility [4]. Disseminated gonococcal infection can lead to septic arthritis and in rare cases septic shock and death [5]. Accurate data regarding the prevalence of sexually transmitted infections in the Georgian military population is currently lacking. The acquisition of such data is essential for the development and deployment of rational intervention strategies [6]. To this end, a collaborative study was initiated between the Georgian Ministry of Defense military hospital in Gori, the US Army Medical Research Directorate in Georgia (USAMRD-G) and the Uniformed Services University of the Health Sciences in Bethesda, Maryland, with the goal of characterizing *N. gonorrhoeae* isolates derived from men being treated for urethritis at the Gori facility. During this study, an unusual isolate of *N. gonorrhoeae* was recovered from a urethral swab taken from a 23-year-old soldier. This isolate displayed resistance to cefixime and ciprofloxacin and reduced susceptibility to penicillin and tetracycline. In order to further characterize this isolate, a detailed molecular and phenotypic analysis was performed. The *N. gonorrhoeae* multiantigen sequence type (NG-MAST) was determined, and the nucleotide sequence of genes associated with vulnerability to the cephalosporins and the fluoroquinolones were determined [7], [8].

Furthermore, the presence of mutations conferring reduced susceptibility to these antimicrobials was uncovered [9], [10], [11], [12], [13], [14]. To our knowledge, this is the first study to describe an *N. gonorrhoeae* isolate containing previously described fluoroquinolone and cefixime resistance mutations from the Republic of Georgia.

## Methods

### Inclusion criteria

All male active-duty service members older than 18 years who presented to the military hospital with the symptoms of acute urethritis were offered enrollment. Acute urethritis was defined as having one or more of the following symptoms: urethral discharge, dysuria, penile irritation or watery, viscous penile secretions.

### Exclusion criteria

Male subjects younger than 18 years of age and female subjects were excluded from the study.

### Laboratory testing

First-void urine and urethral swabs were collected from all patients. Urethral swab specimens were Gram stained and cultured on modified Thayer Martin medium at the military hospital. Cultures were transferred to the USAMRD-G laboratory in Tbilisi for identification and antimicrobial susceptibility testing. Nucleotide sequence analysis of the *gyrA, parC, ponA, penB, mtr* and *penA* alleles was performed at the Uniformed Services University of the Health Sciences in Bethesda, Maryland, using previously published primers [15], [16], [17], [18], [19], [20], [21], as was NG-MAST, which was also performed according to previously published guidelines [20], [22]. Susceptibility testing was performed using Etest (bioMérieux, Hazelwood, MO, USA), and confirmation of the cefixime MIC was done by agar dilution and interpreted using the 2017 guidelines of the Clinical Laboratory Standards Institute [23], [24]. Alignments of predicted amino acid sequences of *penA* alleles was performed using the CLUSTAL 0 (1.2.4) multiple sequence alignment tool [25], [26].

## Case report

A 23-year-old male soldier reported to the military hospital in Gori presenting with symptoms consistent with urethritis. He indicated that he had an acute urethral discharge and that he was experiencing burning sensations during urination. The patient indicated that he had been treated for *Chlamydia* in the past and that he had unprotected sexual contact with a female sex worker at least 10 days before symptom onset. The patient was offered enrollment in the USAMRD-G study and consented. A urethral swab and first-void urine were collected, and he was empirically treated with a combination of ceftriaxone and levofloxacin. The patient was discharged from the hospital and subsequently lost to follow-up. The urethral swab and urine samples were transferred to the Gori hospital laboratory for analysis and culture. Isolates consistent with *Neisseria* sp. were obtained from the urethral swab and forwarded to USAMRD-G for confirmation and characterization.

## Results

Gram stain results from the initial urethral swab indicated the presence of Gram-negative diplococci, and biochemical testing of the resulting isolates revealed that they were both catalase and oxidase positive. These results were found to be consistent with members of the family *Neisseriaceae,* and further analysis was undertaken to confirm these results and to identify the species [17]. Targeted biochemical analysis using a *Neisseria-* and *Haemophilus*-specific panel (API NH; bioMérieux) designed to interrogate bacterial metabolism indicated that the genus and species specific reactions of the isolate were consistent with *N. gonorrhoeae* [18]. Susceptibility testing by Etest revealed that the isolate was resistant to ciprofloxacin and displayed intermediate-level resistance to cefixime, benzylpenicillin and tetracycline (Table 1). The cefixime MIC was also determined by agar dilution and was found to be at a resistant level (MIC 0.5 μg/mL) (Table 1). Molecular typing based on NG-MAST, which targets the *porB* and *tbpB* genes, were consistent with NG-MAST sequence type (ST) 2212 [20], [22]. Intriguingly, gonococcal isolates of ST2212 with reduced susceptibility to the fluoroquinolone class of antibiotics were previously reported in Estonia [19]. The finding of a similar isolate in Georgia may be indicative of a general trend towards reduced fluoroquinolone susceptibility in Eurasian *Neisseria* isolates. Indeed, sequencing of the quinolone resistance-determining region identified three substitutions that are associated with reduced susceptibility to fluoroquinolones (gyrA substitutions S91F/D95G and parC substitution S87R) (Table 2) [9].

**Table 1:** Antimicrobial susceptibilities of *Neisseria gonorrhoeae* isolate obtained from 23-year-old male Georgian soldier

Antimicrobial	Method	MIC (μg/mL)	Interpretation (CLSI)
Tetracycline	Etest	0.75	Intermediate (reduced susceptibility)
Ceftriaxone	Etest	.064	Sensitive
Gentamicin	Etest	3	Sensitive
Benzylpenicillin	Etest	0.5	Intermediate (reduced susceptibility)
Ciprofloxacin	Etest	16	Resistant
Azithromycin	Etest	0.25	Sensitive
Cefixime	Etest	0.125	Intermediate (reduced susceptibility)
Cefixime	Agar dilution	0.5	Resistant
Spectinomycin	Etest	12	Sensitive

Susceptibility data were obtained by Etest and agar dilution methods. MIC values were interpreted using CLSI- and CDC-derived breakpoints [24].

CDC, Centers for Disease Control and Prevention; CLSI, Clinical and Laboratory Standards Institute.

**Table 2:** Antimicrobial susceptibility altering mutations detected in isolate

Gene	Function	Mutation	Potential phenotype
*gyrA*	DNA gyrase	Serine to phenylalanine substitution at position 91 (S91F) and aspartic acid to glycine substitution at position 95 (D95G)	Reduced susceptibility to fluoroquinolones
*parC*	DNA topoisomerase	Serine to tyrosine acid substitution at position 87 (S87R)	Reduced susceptibility to fluoroquinolones
*ponA*	Penicillin-binding protein 1	Leucine to proline substitution at position 421 (L421P).	Reduced susceptibility to penicillins
*mtr*	Multidrug efflux pump	Single base pair deletion (−T/) at −35 site, histidine to tyrosine substitution at position 105 (H105Y) and cysteine to arginine substitution at position 206 (C206R)	Reduced susceptibility to macrolides and β-lactams
*penB* (*porB1b*)	Porin	Glycine to lysine substitution at position 120 (G120K) and alanine to asparagine substitution at position 121 (A121N)	Reduced susceptibility to β-lactams and tetracyclines
*penA*	Penicillin-binding protein 2	53 independent amino acid substitutions	Reduced susceptibility to extended-spectrum cephalosporins

**Fig. 1:**
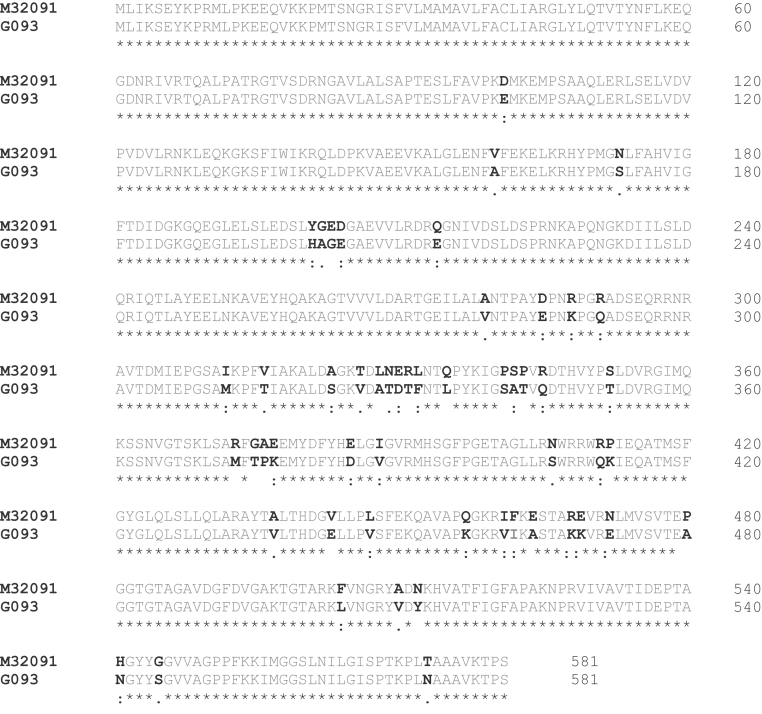
Alignment of predicted amino acid sequences of *penA*-G093 and *penA* wild-type alleles. Fifty-three distinct amino acid substitutions were identified when *penA* wild-type allele was compared to Georgian (G093) allele. Identical amino acids are indicated by asterisk; nonidentical amino acids are indicated by boldface type. Presence of one or two dots below nonidentical amino acids indicates that although wild-type and novel sequence are not identical, they are biochemically and functionally similar [25], [26].

Sequencing of four genes associated with increased resistance to penicillin, *ponA, penB, mtr* and *penA,* was also performed. Analysis of the *ponA* (penicillin-binding protein 1) sequence revealed a leucine to proline substitution (L421P) previously associated with reduced penicillin susceptibility [10]. Glycine to lysine (G210K) and alanine to histidine mutations (A121N) were detected in *penB* (*porB1b*)*.* These mutations have previously been found to reduce susceptibility to β-lactam and tetracycline antibiotics when they occur in the presence of mutations in *ponA* and the *mtr* locus [27], [28]. Importantly, a single base pair deletion (−T/A) was identified in the −35 region of *mtrR.* This deletion is known to decrease expression of the repressor protein MtrR, resulting in increased expression of the MtrCDE efflux pump, leading to decreased susceptibility to macrolides and β-lactams [27]. Furthermore, a mosaic *penA* (penicillin-binding protein 2) allele was also identified [12], [29], [30]. The *penA* allele of this isolate encodes 53 amino acid substitutions and is most closely related to the mosaic *penA* allele XXXIV (Fig. 1) [8].

## Discussion

To our knowledge, this is the first published report of a multidrug-resistant gonococcal isolate identified in the Republic of Georgia. This isolate, named G093, carries a mosaic *penA* allele and other key mutations linked to antibiotic resistance that have not been previously reported in Georgia, including the S91F and D95G mutations in *gyrA and* a S87R mutation in *parC.* The *penA* XXXIV allele is associated with reduced susceptibility to extended-spectrum cephalosporins, but not ceftriaxone resistance [8]. F89 is a ceftriaxone-resistant strain isolated in France that encodes the *penA* allele CI, which is nearly identical to *penA* XXXIV but includes an A501P substitution resulting from a point mutation at nucleotide 1501 [12], [29]. Because residue 501 has been shown to be critical for ceftriaxone resistance, it is possible that the acquisition of one additional point mutation by strain G093 at nucleotide 1501 would yield a novel ceftriaxone-resistant Georgian isolate. The undetected development of such isolates among commercial sex workers would be a significant event and could possibly initiate the end of the utility of the expanded-spectrum cephalosporins as treatment options for gonorrhoea in Georgia.

The results presented in this study are of regional significance given that little is known about the aetiology of sexually transmitted infections and the mechanisms of antibiotic resistance in Georgia. Our results are of broad significance given that the infection was most likely acquired from a sex worker, as sex workers often engage in sexual activities with multiple partners, increasing the chance that organisms with these mutations have spread throughout the country and beyond. Therefore, the identification of the isolate reported here may indicate the potential emergence and spread of a fluoroquinolone- and cephalosporin-resistant *N. gonorrhoeae* strain in Georgia. This report underscores the need for identification and susceptibility testing to be conducted as part of the treatment algorithm for suspected *N. gonorrhoeae* infections. Empirical treatment without susceptibility testing can lead to the emergence of strains with reduced susceptibility and decrease the efficacy of currently available antimicrobial agents [12], [30], [31]. Further research will be required to determine the degree of antimicrobial resistance in the Georgian *N. gonorrhoeae* population, the geographic spread of antimicrobial resistance and the most common resistance mechanisms. It is recommended that identification and susceptibility testing be conducted on all isolates obtained from patients experiencing acute urethritis, particularly when gonorrhoea is suspected.
